# Effects of a psychoeducational intervention in family caregivers of people with Alzheimer’s disease

**DOI:** 10.1590/S1980-57642011DN05030011

**Published:** 2011

**Authors:** Cinthia Costa Ponce, Tiago Nascimento Ordonez, Thaís Bento Lima-Silva, Glenda Dias dos Santos, Luciane de Fátima Viola, Paula Villela Nunes, Orestes Vicente Forlenza, Meire Cachioni

**Affiliations:** 1 Graduate student in Gerontology, School of Arts, Sciences and Humanities of the University of São Paulo, São Paulo SP, Brazil;; 2 Bachelors in Gerontology, School of Arts, Sciences and Humanities of the University of São Paulo, São Paulo SP, Brazil;; 3 Bachelors in Gerontology, Post-graduate in Neurosciences from the ABC School of Medicine, Santo André Foundation, Santo André SP, Brazil and Masters Student in Neurology at the University of São Paulo, School of Medicine, São Paulo SP, Brazil;; 4 Masters in Psychiatry from the University of São Paulo, School of Medicine, São Paulo SP, Brazil;; 5 PhD in Biological Sciences, Medicine Modality from the Institute of Psychiatry of Hospital das Clinicas, University of São Paulo, School of Medicine, São Paulo SP, Brazil;; 6 Masters and MD in Medicine at the Department of Psychiatry of the University of São Paulo. Full Professor at FMUSP. Associate Professor of the Department of Psychiatry of the University of São Paulo, School of Medicine. Vice-Director of the Laboratory of Neurosciences and Head of the Out-patient Unit of Geriatric Psychiatry of the LIM27 Medical Laboratory, Department and Institute of Psychiatry of the FMUSP, São Paulo SP, Brazil;; 7 Professor PhD in Gerontology at the State University of Campinas and Lecturer at the School of Arts, Sciences and Humanities of the University of São Paulo. Head of Psychoeducational Intervention Group for Caregivers of Elderly with Alzheimer’s Disease of the Rehabilitation Center and Day-care Hospital for the Aged within the Institute of Psychiatry of the Hospital das Clínicas of the University of São Paulo, School of Medicine, São Paulo SP, Brazil.

**Keywords:** Alzheimer’s disease, family caregivers, psychological and educational intervention

## Abstract

Psychoeducational activities are a way of promoting help for caregivers of patients with Alzheimer’s disease, representing a forum for knowledge sharing, and in which the primary focus is on psychological themes aimed at carers developing coping skills and strategies.

**Objective:**

The main objective of this study was to gauge perceptions about care and its impact among family caregivers of patients with AD participating in a psychoeducational group intervention, as well as the possible positive and negative aspects associated with this role. The subjective impact of AD on the lives of these caregivers was assessed on each of the dimensions of the Caregiver Burden Scale using a semi-directed interview on perceptions about care.

**Methods:**

This was a prospective study, in which information was collected twice, before and after, psychoeducational intervention. Through the application of the scale, benefits were evident for all dimensions assessed in the instrument (general strain, isolation, disappointment, emotional involvement and environment).

**Results:**

The results showed that after the psychoeducational intervention, caregivers felt less burdened by care compared to pre-intervention. Conclusion: These findings confirm that expanded implementation of psychoeducational interventions for caregivers of patients with AD can be beneficial for both caregivers and patients.

## Abstract (pt)

Atividades psicoeducacionais constitui um meio de promover auxílio a cuidadores de acientes com Alzheimer’s disease (DA), representam um espaço para o compartilhamento de conhecimentos que tem como foco primário temas dirigidos a estratégias e habilidades de enfretamento.

**Objetivo:**

O objetivo do presente estudo foi levantar as percepções sobre o cuidar e seu impacto entre cuidadores familiares de portadores de DA, participantes de um Grupo de Intervenção Psicoeducativa. Visou-se também detectar possíveis impactos em variáveis objetivas e subjetivas, pré e pós intervenção psicoeducacional.

**Métodos:**

Esta pesquisa tratou-se de um estudo prospectivo, no qual foram coletadas informações em dois momentos distintos, antes e após a intervenção psicoeducativa. Através da aplicação da Escala perceberam-se benefícios em todas as dimensões envolvidas nesta (tensão geral, isolamento, decepção, envolvimento emocional e ambiente).

**Resultados:**

Os resultados demonstraram que após a intervenção psicoeducativa, os cuidadores apresentaram menor sobrecarga em relação ao cuidado, do que comparado ao início da intervenção.

**Conclusão:**

Esses achados confirmam que as intervenções psicoeducacionais implementadas de modo expandido podem ser benéficas para cuidadores e pacientes com DA.

Brazil is experiencing a demographic shift characterized by lower fecundity rates, lower mortality and accelerated urbanization, all of which have contributed to a growth in the proportion of elderly compared to other age groups.^1^ The increase in life expectancy can imply improved quality of life for some, but it also can signify for others living with chronic degenerative diseases, disabilities and dependency.^2,3^ Among the health problems afflicting the elderly, dementia has the greatest impact on the individual, family structure and society.^4-8^

Alzheimer’s disease (AD) is the most common dementia etiology (in around 50-70% of cases)^9-11^ and besides its consequences on the affected individual, it also has a major impact on everyday living of families, placing an emotional burden on the whole family unit.^12^ AD can cause changes in family structure and roles.^13,14^ Children become caregivers of their demented parents, looking after them and taking on the duties of carer – a situation which may create conflict if not handled properly.^15^

With the progression of dementia, patients become progressively more dependent on caregivers.^16,17^ Caregivers can be classified into formal and informal types. Informal caregivers include family members who intuitively contribute to caring whereas formal caregivers are paid care professionals. The latter have emerged as part of a social movement that sought to create a new mindset able to embrace old age by fostering a new subjectivity in which aging is perceived in a more constructive light. The construction of this new mentality proposes that this embracing of the elders start whenever possible at the family level.^18^ Informal caregivers are those who care for the elderly person voluntarily without remuneration, and they may be neighbors, members of the community, religious groups, friends, and/or relatives (i.e. family caregivers).^19^

Brazilian studies aimed at better understanding domiciliary care have highlighted the heterogeneity of the caring process and point out that family caregiving is influenced by several factors. The life story of family members, the native culture and historical and cultural setting, availability of personal and social support resources, all number important factors. Similarly, family relationships, specificities and heterogeneity of the time and situation pertaining to caregiving, the type and degree of care needs of the elder, the prevailing family arrangements and quality of family relationships should all be taken into account.^20^

A study performed by Karsch^21^ about caregivers of dependent elderly revealed valuable data on caregivers: in 98% of cases examined, the caregiver was a family member, and of these, 92% were female. The majority constituted spouses (44.1%), daughters (31.3%) followed by sisters and daughters-in-law while 67.9% of caregivers provided care without any external help.

The diagnosis of AD is a major event for the family, since from this point forward they are subject to a barrage of feelings: hope for a cure; electing of the caregiver; the financial issues regarding the high cost of drug-based treatment; and the search for self-help.^22^ The diagnosis of the disease can have a huge impact on the family, leading to fear of the unknown and what the future may bring.^23^ Family assistance is extremely valuable and often of major importance in successful treatment and care of the patient.

Amid reports in the literature of the difficulties and stress experienced by caregivers related to performing their everyday tasks, implementation of support or self-help groups has become an increasingly popular strategy adopted by socio-educational and health services to assist those who face the same or similar problems.^8^ Psychoeducational activities are a way of promoting help for caregivers, representing a forum for knowledge sharing, and in which the primary focus is on psychological themes aimed at carers developing coping skills and strategies.^24^ The goals of these efforts are educational, prevention and/or to promote psychological health. In general, psychoeducational interventions are aimed at educating individuals involved in situations with a high risk of developing psychopathological symptoms or to help those facing normative life events (e.g. retirement) or non-normative events (e.g. caring for a relative with AD).^24^

Studies have reported that greater knowledge and awareness about a disease or stressing situation, and of its implications for one’s life and that of others, increases the feeling of control and elicits more effective coping strategies. Therefore, psychoeducation does not comprise a given treatment but rather an approach, which can be used as an adjunct to psychotherapeutic or biomedical therapies.^24,25^ Psychoeducational groups resemble conventional classes but include counseling groups. Nevertheless, the emphasis is on education or learning rather than self-awareness or self-understanding, although these elements are involved.^26^

Psychoeducational groups can include individuals directly affected by a given event or family members and caregivers involved in the process such as:^26^

- Training on social skills for individuals who lack assertiveness;
- Memory training in aging;
- Relatives of patients with schizophrenia;
- Depressive disorders (depressive individuals and their families);
- Formal and informal caregivers of patients with Alzheimer’s disease.

One of the goals of psychoeducation is to prepare family members to monitor the course of the disease and to alert professionals of relapse. Closer monitoring of disease treatment and symptoms can have major implication in the evolution of the disease over the long term.^27^

Group sessions can help reduce feelings of family isolation and provide an opportunity for family members to share experiences and find some comfort in the knowledge that they are not striving alone. The sessions are also conducive to less formal interaction between families and healthcare professionals.^27^

The activities carried out by the Rehabilitation Center and Senior Day-care Hospital (CRHD) of the Institute of Psychiatry at the Hospital das Clinicas - University of Sao Paulo Medical School aim to accompany elderly with mild to moderate AD and their caregivers and/or relatives.

Cognitive rehabilitation for elderly with Alzheimer’s Disease is not a compulsory part of the treatment but according to Abrisqueta-Gomez,^28^ recent studies of patients at the early to moderate stages of dementia have shown that treatment using basic medicines allied with cognitive rehabilitation interventions can help stabilize, or even slightly improve, cognitive and functional deficits, with a consequent reduction in behavioral problems.

These group interventions involving caregivers seek to perform group-based, reflective and educational activities. The activities carried out aim to promote improvements in the quality of lives of patients and caregivers alike and to assist in the demands and needs arising due to the disease.

The target group of this study was the Psychoeducational Intervention for caregivers of patients with AD Group, with participating members of a multi-professional program boasting a multi-disciplinary team enabling provision of a global service catering for the needs of the families and patients. By using a non-pharmacological approach, this program aims to rehabilitate elderly with mild to moderate AD and to provide care aimed at their formal and informal carers through weekly meetings held over a four-month period. Based on group dynamics, video debates on AD, discussion about articles and documentaries on a range of aspects of AD, the act of caring, open dialogue, expressing of emotions, doubts and difficulties, and the sharing of experiences, the group reflects on strategies to cope with day-to-day problems.

The aim of the present study was to gauge the perception on caring and its impact among family caregivers of AD patients taking part in a Psychoeducational Intervention Group. The study also sought to detect possible impact on objective and subjective variables, pre and post psychoeducational intervention.

## Methods

A prospective study was carried out gathering information at two timepoints: before and after a psychoeducational intervention. Caregivers of patients with AD who participated in the Psychoeducational Intervention Group from the Institute of Psychiatry at the Hospital das Clinicas - University of Sao Paulo Medical School for one semester were invited to take part in this study and included upon acceptance. Individuals who discontinued participation in the group during the study period were excluded from the study.

### Venue for psychological interventions

The meetings took place at the Institute of Psychiatry at the Hospital das Clinicas - University of Sao Paulo Medical School. The Psychoeducational Intervention for caregivers of patients with AD Group met on a weekly basis in order to learn about the process of the disease and the different facts concerning care. The group represented a forum for mutual learning, fostering a support network to cope with the disease process and seeking improvements in the emotional welfare of caregivers.

During each meeting, a theme relevant to the disease process was addressed, leading on to open dialogue for questions, reflections, sharing of experiences on ways of dealing with the day-to-day problems of caring. The caregivers expressed their anxieties and fears, outlining the huge emotional strain they are under.

In addition to the intervention involving formal and informal caregivers of patients with early to moderate stage AD, the group also carries out research and fosters human resource training in Gerontology.

As depicted in Chart I, the content of the sessions was divided into five axes of knowledge: Brain and the dementia process; Dementia of the Alzheimer type; Pharmacological and non-pharmacological treatment; Physiological and behavioral changes affecting day-to-day activities; and Care.

**Chart I:** Axes and contents.

Axes	Content
1. Brain and dementia process	• What is the brain? • Senescence × Senility. • Reporting experiences.
	• What is dementia? • Most common dementia types. • Reporting experiences.
2. Alzheimer type dementia	• Alzheimer’s disease: causal factors and importance of early detection. • History of discover and main advances in current research. • Reporting experiences.
	• Alzheimer’s disease. • Changes in the brain and stages of the disease (focus on initial and moderate phases • in line with Group structure). • Reporting experiences.
	• Alzheimer’s disease and world reality – video presentations. • Importance of information about the disease. • Reporting experiences.
3. Pharmacological and non-pharmacological treatment	• Treatment: Pharmacological intervention and cognitive rehabilitation / What results • are expected in initial and moderate stages? • Importance of multi-professional team. • Reporting experience.
4. Physiological and behavioral changes reflected in everyday life	• The day-today lives of AD patients (I) / What to expect in initial and moderate • stages? (ADLs and IADLs – private aspects / ergonomics – inclination for mobility • and risk situations; restroom, wardrobe (personal care), kitchen). • Resolving problems for each demand. • Regularity of life style during phases. • Reporting experiences.
	• Day-today lives of AD patients (II) / What to expect in initial and moderate stages? • (activities/leisure, direction, financial management, external risk situations). • Resolving problems for each demand. • Regularity of life style during phases. • Reporting experiences.
	• Nutritional aspects and the importance of physical activity. • Reporting experiences.
5. Care	• Cine debate: Clips from film Iris. • Reporting experiences.
	• Reporting experiences.Cine debate: Short film Clarita. • Reporting experiences.
	• Reporting experiences.Familial care and formal care. • Reporting experiences. Formal care – What are the professional demands? • Reporting experiences.
	• Reflection on care. • Stress management techniques. • Reporting experiences.

### Instruments

Semi-directed interview consists of an instrument comprising open and closed questions intended to collect sociodemographic data on both patient and caregiver, and questions on the carer/elder relationship and the perception regarding the role of caregiving.

Caregiver Burden Scale^29,30^ (Translated, adapted and validated Brazilian version) is an instrument comprising 22 questions, grouped into five dimensions (General strain, Isolation, Disappointment, Emotional involvement and Environment). The scale enables obtention of a global score and scores on each of the dimensions. These instruments “showed good indices of reproducibility and validity in our milieu, proving a useful instrument for measuring subjective impact of chronic diseases on caregivers”.^31^

### Procedures

Individual interviews lasting approximately 40 minutes were conducted and the proposed instruments applied, after caregivers had read and signed the Free and Informed Consent Term.

### Statistical analyses

The information obtained from the instruments were submitted to univariate and bivariate descriptive statistical analysis. In order to describe the sample profile according to the several study variables, frequency tables of categorical variables, and descriptive statistics, such as measures of position and dispersion of continuous variables, were built.

All continuous variables of interest in this study were submitted to the Shapiro-Wilk test, revealing the absence of a normal distribution and the need for non-parametric tests. Wilcoxon’s test was used to compare non-parametric, repeated measures or dependent variables. The Kruskall-Wallis test was employed to compare scores on the five domains of the Caregiver Burden Scale. Spearman’s correlation coefficient was used to analyze the relationship among numeric variables. Values close to +1 indicated strong correlation among values whereas values close to 0 showed an absence of any relationship among the variables.^32^

The internal consistency of the Caregiver Burden Scale was analysed by calculating Cronbach’s alpha coefficient. On Cronbach’s alpha test, values of 0.80 are taken to indicate high internal consistency and values between 0.60 and 0.79 as intermediate consistency.^32^

The data were keyed into the SPSS 18 Program for later analysis using the *Statistica* 7.0 (2004) software program, The level of significance adopted for the statistics tests was 5%, corresponding to a p-value<0.05

### Ethics aspects

The present project was submitted to the Research Ethics Committee of the Institute of Psychiatry at the Hospital das Clinicas - University of Sao Paulo Medical School. Every participant signed two copies of the free and informed consent term (one of which was to be retained by the participant) thereby guaranteeing the right to voluntary participation and withdrawal from the study.

## Results

The present study was performed in a sample comprising 17 caregivers of patients with AD. Table 1 depicts the sociodemographics of the caregivers. A total of 70.6% of the caregivers were women, and 41.2% were married.

**Table 1:** Care profile among caregivers and elderly with Alzheimer's disease.

Care profile	(n)	(%)
Kinship with person cared for		
Niece/Nephew	2	11.80
Formal caregiver	5	29.40
Grandchild	1	5.90
Son/Daughter	2	11.80
Spouse	6	35.30
Companion	1	5.90
Cared for other person		
Yes	7	41.20
No	10	58.80
Share the care		
Yes	7	41.20
No	9	52.90
No response	1	5.90
Live with elder		
Yes	10	58.80
No	7	41.20
Time living with elder		
Up to 5 years	7	41.20
From 6 to 10 years	2	11.80
From 11 to 20 years	0	0.00
More than 21 years	7	41.20
No response	1	5.90
Time as caregiver		
Up to 5 years	15	88.20
From 6 to 10 years	0	0.00
From 11 to 20 years	0	0.00
More than 21 years	1	5.90
No response	1	5.90
Change in family financial situation		
Yes	6	35.30
No	10	58.80
No response	1	5.90
Move of another person into domicile due to AD		
Yes	6	35.30
No	10	58.80
No response	1	5.90

In terms of the care profile, 58.9% of caregivers had never cared for another person, and 52.9% did not share the task of caring with any one (Table 2).

**Table 2:** General data on elderly patients with AD.

Disease	(n)	(%)
Disease stage		
Mild	9	52.90
Moderate	8	47.10
Advanced	0	0
Terminal	0	0
Time since diagnosis		
Less than 1 year	2	11.80
1 year	3	17.60
2 years	7	41.20
3 years	2	11.80
4 years	1	5.90
5 years or longer	1	5.90
No response	1	5.90
Holds private health insurance		
Yes		94.10
No		5.90
Diagnosing specialist		
Geriatrician		47.10
Psychiatrist		11.80
Neurologist		41.20
No response		5.90

With regard to disease stage, 52.9% were at a mild stage of the disease while 47.1% were at a moderate stage. No patients were at other phases, since as the disease progresses limitations become greater due to the intensity of symptoms.

**Figure 1:**
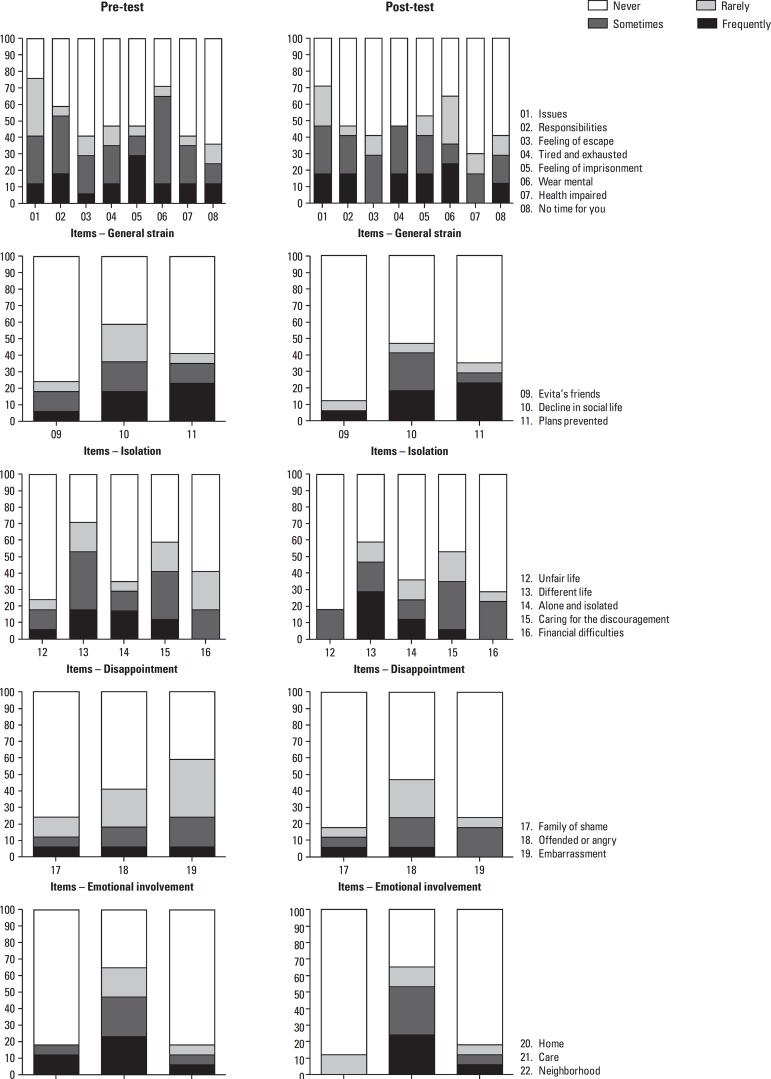
Distribution of scores for items and domains of Caregiver Burden Scale.

**Figure 2:**
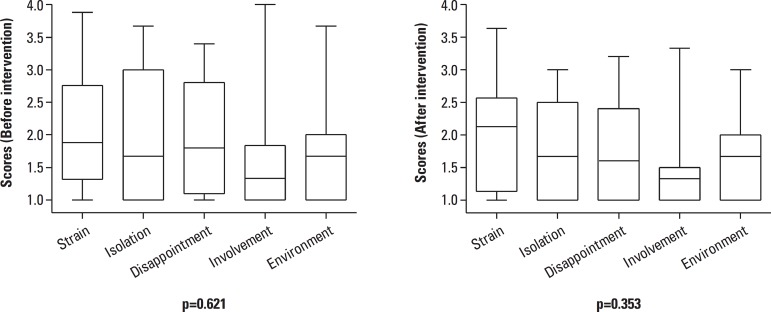
Tendencies in distribution of caregiver scores by domain and time on Caregiver Burden Scale. Results of Kruskall-Wallis tests among domains, pre- and postintervention, shown under each box blot.

Figure 1 shows the distribution of scores for the items and domains from the Caregiver Burden Scale. Table 4 depicts the results on the Wilcoxon test for related samples, which compared the total and domain scores of the Caregiver Burden Scale, before and after the group psychoeducation intervention. Figure 2 shows the tendencies of distribution of scores of caregivers, by domain and time, on the Caregiver Burden Scale, as well as the results on the Kruskal-Wallis tests among domains, before and after the group psychoeducation intervention.

**Table 4:** Comparison of study results showing mean global values on Caregiver Burden Scale applied in caregivers of patients with Alzheimer's disease.

Dimensions	Mean result - Before	Mean result - After	Mean global values
General Strain	2.07	1.99	2.39
Isolation	1.86	1.73	2.28
Disappointment	2.00	1.78	2.22
Emotional Involvement	1.65	1.51	1.72
Environment	1.73	1.63	1.90
Global	1.92	1.79	2.18

As evident in Figure 1, the domains show no improvement in general score, which leads us to generalizing that the information obtained in the psychoeducational group had a positive impact on the caregiver.

On the General Strain domain, scores for responses tended to be greater (Figure 2), in other words, there was a higher number of negative answers. A hypothesis for this finding may be that, upon learning about the disease and its future, caregivers felt deeper concern. However, on some questions there was a reduction in burden and negative feelings, such as for questions 3 and 7 on which caregivers had initially given the answer “often” during pre-test but not at post-test. Overall, a positive impact was seen on this domain and the subdomains assessed.

For the Isolation domain, a large increase in the answer for question 9 “Not at all” was observed. Answers tended to be associated to lower scores. On the Disappointment domain, improvement was seen mainly on questions 12, 15 and 16. Similarly to the Isolation domain, there was a tendency toward lower scores in the results. With regard to the Emotional Involvement domain, an improvement was seen mainly on questions 17 and 19, where the latter was not answered with “often” at post-test. This domain scored less difference on post-test compared to the other domains and to results at pre-test. Concerning the Environment domain, an improvement was seen mainly on question 20, initially answered with “sometimes” and “often” yet not on post-test.

As shown in Table 3, the means of all the domains for responses of caregivers were lower, with the Disappointment domain showing the greatest reduction. The negative delta for the mean and median show fewer negative answers after the psychoeducational intervention and the reapplication of the Caregiver Burden Scale.

**Table 3:** Results on Wilcoxon tests for related samples comparing total and domain scores on Caregiver Burden Scale, before and after psychoeducation intervention.

Variables		Descriptive statistics					*p-value*
		**Mean**	**SD**	**Minimum**	**Median**	**Maximum**	
General strain	Before After Delta	2.07 1.99 -0.08	0.94 0.89 0.69	1.00 1.00 -1.75	1.88 2.13 0.00	3.88 3.63 0.88	0.755
Isolation	Before After Delta	1.86 1.73 -0.14	0.94 0.83 0.51	1.00 1.00 -1.33	1.67 1.67 0.00	3.67 3.00 1.00	0.272
Disappointment	Before After Delta	2.00 1.78 -0.22	0.88 0.74 0.71	1.00 1.00 -2.40	1.80 1.60 -0.20	3.40 3.20 0.80	0.279
Emotional involvement	Before After Delta	1.65 1.51 -0.14	0.82 0.76 0.50	1.00 1.00 -1.33	1.33 1.33 0.00	4.00 3.33 0.67	0.290
Environment	Before After Delta	1.73 1.63 -0.10	0.77 0.58 0.79	1.00 1.00 -1.67	1.67 1.67 0.00	3.67 3.00 1.67	0.721
Global	Before After Delta	1.92 1.79 -0.13	0.74 0.65 0.45	1.09 1.00 -0.95	1.77 1.77 -0.09	3.59 2.91 0.59	0.309

Table 4 provides a comparative analysis of values obtained in this study set against findings of other investigations for caregivers of patients with AD in different locations worldwide. Notably, the values for the present study across all dimensions, both before and after the intervention, were lower than those reported worldwide.

## Discussion

The typical profile of caregivers of patients with AD, as found in the literature, is: female, family member, residing at the same domicile, and in general a daughter or wife.^33^ The findings for the present study resembles this profile since the majority of caregivers were women (70.6%), spouses (35.3%), and residing at the same domicile as the AD patients (58.8%). This finding reflects a cultural and social pattern in which the role of caregiving is seen as a female duty.^34-36^

The present investigation also showed a number of grandchildren who were caregivers. The study by Brody^37^ noted that this grandparent-grandchild relationship could be beneficial for the AD patient but may have a negative impact in terms of social limitations experienced by the grandchild caregivers.^38^ Also, a greater number of caregiver nephews/nieces than reported in the literature was found, perhaps demonstrating greater concern by these relatives over their uncles/aunts who have no children, and therefore feeling responsible for them.

With regard to the profile of patients with AD, most were women (76.5%), aged 80 years or older (52.90%), followed by those in the 70 to 79 year age group, indicating that proportion increases with age. In terms of marital status, the majority of the individuals were married (47.10%) or widowed (41.2%). These data corroborate those found in the literature reported by Fernandes and cols.^39^ who affirmed that advanced age and female gender constitute risk factors for AD.^40^

In the family context, the person assuming the role of caregiver is subject to care demands, which affects them physically, mentally and socially. According to the literature, caregivers are middle-aged and elderly women who perform the activity in compliance with cultural norms in which they are held responsible for organizing family affairs, caring for the children and elderly. The present study corroborated this knowledge.^40^

In relation to the schooling variable, caregivers had a heterogeneous educational level albeit similar to the schooling of the elderly with AD. This contrasts with other studies showing caregivers to have a higher level of schooling than the elderly.^40^

Concerning the care activity itself, most caregivers had never cared for another person, and did not share the task of caring with another. This fact may give cause for concern since the task of caregiving is exhausting and represents a risk factor for carer health.^41^

Another factor influencing the fact that only one family member was responsible for caring was that the majority of caregivers lived with the AD patient. A large proportion had lived with the patient for over 21 years, although had taken on the role of caregiver only fairly recently (less than 5 years earlier) making them relatively inexperienced at the task.

The task of caring for an elderly patient with AD demands almost constant commitment from the caregiver, who must sacrifice their habitual activities in order to perform the role. Some carers give up their jobs or professions and stop living their own lives, often leading to social isolation and depression. Studies show that the caregiver, upon taking on the care of the old person in the home, frequently manifests their discomfort and feeling of loneliness when they do not feel they are getting any support from other members of the family.^42,43^ The need to share the fatigue caused by the constant exposure to negative events indicates the wish to soften the impact caused by the burden imposed by the caring activities or otherwise. It is essential caregivers receive the support they need from other members of the family, even if this takes place during visits, since prolonged exposure to a potentially stressing situation strongly contributes to overall burnout of the individual and the feeling of burden as a result of the psychosocial effects of the disease.^40,42^

Providing care for a partially dependent individual inevitably leads to change in the life style of the carer in order to cater for the needs of the patient. Independently of the age of the carer, their leisure time activities and social life become altered, giving the impression that they no longer have the autonomy to manage their own life and are living to serve the needs of another. The individual requiring care on the other hand, ends up demanding the presence of the caregiver and does not always react well to their absence.^44^

According to Mendes,^43^ the lack of freedom and the lonely times spent by the caregivers make them and the elderly person embark on a quest, striving to rebuild and reestablish, in a bid to return to pre-morbid routines, a feat made impossible due to the need for care and the dependence of the elder on the caregiver. The process of rebuilding the life of the carer is one of conflict, since it takes time to rework new routines, activities, personalities, and identities born of discontinuity, yet hinges largely on each individual’s life history.^43,44^

The finding that majority of the family members reported no financial impact is a positive one since the financial burden of AD represents yet another stressor in the gamut of tasks carried out by the caregiver, since they must manage the elder’s financial affairs in addition to their own. This explains why caregivers often calculate their income together with that of the elder in order to facilitate the management of resources.^40^

Amid efforts to adjust their lives, primary caregivers also face problems since, in the absence of secondary carers, the elder often ends up on their own at the home while the carer performs their activities outside the home - a situation which generates concern for the carer. With time, the caregiver is able to redefine their role in terms of daily tasks and care activities, which although strained, enables them to envisage leisure options and forms of relaxation. In order not to fall sick themselves, caregivers must realize that they have a life and are not ailing, and must carry on their lives as best they can.^44^

With regard to phase of the disease, the majority of patients were at a mild to moderate stage, during which diagnoses are often underway and upon confirming the disease, there are not so many limitations arising thereafter with advance of the disease for travelling to seek assistance from other professionals outside the domestic setting. Regarding time since diagnosis, results showed that the vast majority had been diagnosed within the last two years, showing that family members had sought help after confirmation of the disease.

The vast majority of patients had private health care insurance, rendering it easier to seek professional help. With regard to the diagnosis, majority were reached by geriatricians, professionals qualified to confirm disease diagnosis since they are familiar with the aging process and can monitor other comorbidities of the patient besides the advance of AD, as well as reduce chances of drug interactions for instance, by virtue of knowing the patient’s medical course.

On the Caregiver Burden Scale, statistical data indicated a scale coefficient of 0.93 for the first application and 0.92 at the second interview, demonstrating high internal consistency of the instrument and validity for the subsequent results on the domains.

Based on the scale, the psychoeducational intervention yielded benefits for the caregiver in dealing with the disease in their relative with Alzheimer’s disease after the psychoeducational intervention. The problems of caregivers are one of the most significant negative effects inherent to AD.^36,45^ Therefore, assessment of caregivers should be an integral part of treatment for this dementia,^36,46^ since support for the family members and caregivers is paramount.^36-47^ A reduction in caregiver burden across all domains studied was evident following the intervention. Furthermore, for all dimensions, the results were found to be lower in the present study than those reported by other studies investigating mean global values of the scale when applied to caregivers of AD patients.

In order to prevent a stressing family relationship marked by exhaustion of family members owing to frustration which can result in mistreatment and abuse; it is necessary that caregivers have sufficient physical and emotional strength, knowledge about the disease and the ability to deal with the illness, along with other factors such as financial and social strain as well as family conflict.^38^

In terms of Global Strain, the studies revealed that dementia syndromes can generate caregiver strain, since during the course of the disease, the elder becomes progressively more dependent, a situation constituting a source of suffering and feeling of impotence among caregivers. Dementia disorders may destabilize both the elderly and their family members.^49^ The literature reviewed also showed this variable can be associated with the number of tasks undertaken. Evidence shows that the higher the number of activities caregivers undertake, the greater effort needed and consequently the greater the resulting fatigue and physical and mental stress.^50^

For the Isolation dimension, studies highlight that, moving out of social circles and avoiding the presence of friends can lead to a series of health problems in caregivers. The present study corroborates this notion in revealing an association of this dimension with the variables health problems and number of comorbidies.^50^

Concerning the Disappointment dimension, the literature reports no association with the variables related to the caregiver. No extensive studies were available addressing this dimension.

On the Emotional Involvement dimension, the literature shows an association with degree of kinship of caregivers, where spouses suffer the greatest impact upon caring for the demented patient. The study by Pruchno and Resch^51^ shows that caregiver spouses are the most vulnerable to burden. In the present study, caregivers were predominantly wives. This dimension touches on delicate aspects that are difficult for caregivers to address, posing some questions which are hard to answer since in doing so the caregiver is admitting they are ashamed of the behavior of their relative.^50^

In relation to Environment, studies have shown that the family setting, both social and physical, can be unfavorable in the elder/caregiver relationship. In relation to the familial and social environment, studies in the literature consider healthy family and social relations important for relieving the daily pressures and appraising the burden of care provision. With regard to physical environmental conditions, these can cause discomfort for the elder as well as the caregiver who on some occasions needs to make adjustments in order to accommodate the elder which affect the privacy of other family members. Equipment to assist elderly with their mobility problems are quantitatively lacking.^52^

The perception of subjective impact using the CBS depends largely on the way the caregivers appraise their situation. This primary assessment process corresponds to the stress model by Lazarus and Folkman^53^ and described by Lawton et al.^54^ According to Lawton, a stressor event (an illness for example) triggers an appraisal process in the caregiver which defines an external situation as a stressor or non-stressor (primary appraisal). Thus, stressor events can only be assessed in context by taking into account factors linked to the patient, disease, the environment and the family dynamics. The presence of a relative with AD in the family setting is a potentially conflicting situation and driven by constant strains, which directly affect the caregiver and the family dynamics.^50^

To sum up, this study shows that after a psychoeducational intervention, caregivers report less burden (measured by the CBS), compared to pre-intervention. Despite the low number of participants, the results of this study showed improvement on all five domains contained in the scale (general strain, isolation, disappointment, emotional involvement, and environment). These findings confirm that expanded implementation of psychoeducational interventions for caregivers of patients with AD can be beneficial for both caregivers and patients, and since the care of the latter depends on the former, interventions enhance the quality of life of both. A need was identified for a multi-professional and inter-disciplinary team to support the caregivers in assuring they remain in good psychological health to continue their caregiving role. In turn, this highlights the need for highly qualified specialists to assist caregivers as well as patients and for greater investment in studies toward preventing negative effects on caregivers.
